# Identification of Gender- and Subtype-Specific Gene Expression Associated with Patient Survival in Low-Grade and Anaplastic Glioma in Connection with Steroid Signaling

**DOI:** 10.3390/cancers14174114

**Published:** 2022-08-25

**Authors:** Alex Hirtz, Nolwenn Lebourdais, Magalie Thomassin, Fabien Rech, Hélène Dumond, Hélène Dubois-Pot-Schneider

**Affiliations:** 1 Université de Lorraine, CNRS, CRAN, F-54000 Nancy, France; 2 Université de Lorraine, CHRU-Nancy, Service de Neurochirurgie, F-54000 Nancy, France

**Keywords:** glioma, steroid signaling, transcriptomic profiles, gender-specific analysis, data mining, survival analysis, functional enrichment

## Abstract

**Simple Summary:**

Gliomas are primary brain tumors that are initially slow growing but progress to be more aggressive and, ultimately, fatal within a few years. They are more common in men than in women, suggesting a protective role for female hormones. By analyzing patient data collected in the public TGCA-LGG database, we have demonstrated a link between the expression level of key steroid biosynthesis enzymes or hormone receptors with patient survival, in ways that are dependent on gender and molecular subtype. We also determined the genes which expression associated with these actors of steroid signaling and the functions they perform, to decipher the mechanisms underlying gender-dependent differences. Together, these results establish, for the first time, the involvement of hormones in low-grade and anaplastic gliomas and provide clues for refining their classification and, thus, facilitating more personalized management of patients.

**Abstract:**

Low-grade gliomas are rare primary brain tumors, which fatally evolve to anaplastic gliomas. The current treatment combines surgery, chemotherapy, and radiotherapy. If gender differences in the natural history of the disease were widely described, their underlying mechanisms remain to be determined for the identification of reliable markers of disease progression. We mined the transcriptomic and clinical data from the TCGA-LGG and CGGA databases to identify male-over-female differentially expressed genes and selected those associated with patient survival using univariate analysis, depending on molecular characteristics (IDH wild-type/mutated; 1p/19q codeleted/not) and grade. Then, the link between the expression levels (low or high) of the steroid biosynthesis enzyme or receptors of interest and survival was studied using the log-rank test. Finally, a functional analysis of gender-specific correlated genes was performed. *HOX*-related genes appeared to be differentially expressed between males and females in both grades, suggesting that a glioma could originate in perturbation of developmental signals. Moreover, aromatase, androgen, and estrogen receptor expressions were associated with patient survival and were mainly related to angiogenesis or immune response. Therefore, consideration of the tight control of steroid hormone production and signaling seems crucial for the understanding of glioma pathogenesis and emergence of future targeted therapies.

## 1. Introduction

Diffuse low-grade gliomas (LGG) (or grade 2 gliomas in the 2021 WHO classification; [1]) are rare brain tumors of glial origin (about 15% of gliomas) diagnosed in young adults (median age 40 years). They classically evolve in two phases: the first, corresponding to slow growth and invasion, indolent during several years before and after the radiological diagnosis, and the second, rapid phase related to anaplastic transformation (histological grade 3 or 4), leading to neurological disorders and ultimately to the death of patients (median overall survival 15.7 years) [2]. One of the main issues for cancer biologists remains the finding of reliable markers to help the follow-up of disease progression, especially during the anaplastic transformation.

Although their management is currently not standardized, the general aim of treatment is to delay this anaplastic transformation (which remains almost inevitable). Surgery plays a key role in decreasing tumor volume, significantly impacts the course of the disease, and improves patient survival, while preserving the quality of life [3]. Alkylating agent-based chemotherapy tends to play an increasing early role, before or after surgery, while radiotherapy has the same impact whether it is delivered early or late. Therapeutic schemes remain very similar for every patient, although, according to the 2021 WHO classification, gliomas split into three types: IDH1-mutated/1p/19q-codeleted oligodendroglioma, IDH1-mutated astrocytoma, or IDH1 wild-type glioblastoma [1]. Therefore, molecular, cellular, or physiological determinants of this heterogenous disease that would drive precision therapy or report individual response to treatment are still urgently needed.

The etiology of low-grade gliomas remains unknown. The tumor develops during adolescence or in young adults and is often diagnosed after the occurrence of an epileptic seizure (70–90%). The incidence and poor outcome of these tumors appear to be higher in men than in women [4]. Since no cellular model is currently available to decipher the underlying molecular and cellular differences, Khan et al. (2021) recently mined public databases to identify gender-specific molecular differences in transcriptomic and epigenomic datasets, mixing low-grade 2 and anaplastic grade 3 gliomas from some databases, including IDHwt and IDHmut samples [5]. From 1564 transcripts located on autosomes, ontology analyses revealed that these genes were mainly related to cell–cell junction, communication, angiogenesis, and immune system processes. However, their reliability as prognosis factors to predict patient survival and consequently refine glioma classification for precision medicine remains to be established, depending on the molecular status (IDH wild-type/mutated; 1p/19q codeleted/not) and histological grade of the tumor.

Other factors appear to influence the natural history of gliomas, including steroid hormones. In synergism with progesterone, the balance of estrogens and androgens exerts important modulator effects on brain development, including neuronal excitability and connectivity and neoplastic glial cell proliferation control. A recent review from Hirtz et al. (2020) highlighted the “female paradox” that exogenous estrogen exposure might protect women against gliomagenesis, whereas endogenously produced estrogens or progesterone could promote neoplastic transformation and tumor growth [6]. In males, high levels of circulating androgens were associated with increased glioma risk and poorer outcome [7,8]. In case of pregnancy, the deleterious effect of hormone levels appeared more striking: eight cases of pregnant LGG patients were initially reported, six of which had a negative impact of pregnancy i.e., accelerated tumor growth. Then, the series was extended to 11 pregnant LGG patients for 12 reported pregnancies and supported the increase in tumor growth velocity during pregnancy. Postpartum growth rate recovered to pre-pregnancy level [9]. Finally, Peeters et al. (2018) confirmed in a series of eight glioma patients that pregnancy accelerated the tumor growth rate by almost seven-fold and led to clinical deterioration [10]. Despite the small number of cases studied, those data suggested that the burst in estrogen/progesterone production observed during the second part of gestation might trigger a low-grade tumor growth increase and/or anaplastic transformation.

The key actors of the steroid signaling include biosynthesis enzymes such as Cytochrome P450 Family 19 Subfamily A Member 1 (*CYP19A1* encoding aromatase), which converts androgens to estrogens and hormone receptors. Indeed, the effects of androgens, estrogens, and progesterone are mediated by the nuclear receptors, AR (encoded by the *AR* gene), ERα (encoded by the *ESR1* gene), or ERβ (encoded by *ESR2*), respectively, and progesterone PR (encoded by *PGR*), as well as by the g protein-coupled membrane receptor *GPER1*. The *ESR1*, *ESR2*, and *PGR* genes prognostic values begin to be well-described in glioblatoma (GBM) but remain unknown in low-grade and anaplastic tumors. Nevertheless, ERα and ERβ mRNA decrease, whereas PR expression increases with the histological grade of the tumor [11,12]. A positive association between ERα and ERβ and survival was also reported in gliomas [12]. No data are currently available on the involvement of *GPER1* in low-grade gliomas.

In this paper, we analyzed the clinical and transcriptomic data available at the TCGA-LGG and CGGA databases, which collect samples of low-grade (grade 2) and anaplastic (grade 3) gliomas, and stratified the patients according to gender, tumor grade (2 or 3), and molecular features indicated in the WHO 2016 classification (IDH wild-type/mutated; 1p/19q codeleted/not). First, we analyzed the molecular processes underlying G2 to G3 transformation to clarify which mechanisms might be involved in accelerated tumor growth during pregnancy. For each group, we then selected the (male over female) differentially expressed genes (DEGs) and determined the impact on survival of the expression of key actors of steroid signaling. Finally, to infer the mechanisms involved in the gender-based differences in each grade 3 glioma type, we performed a gene ontology analysis on the genes whose expression was correlated with that of survival-associated steroid genes. This study brings new light in the knowledge of grade 2 and 3 gliomas by highlighting gender- and tumor-type-specific mechanisms involving angiogenesis and immune response.

## 2. Materials and Methods

### 2.1. Data Collection from the TCGA and CGGA Databases and Normalization

Transcriptomic and clinical data from patients with grade 2 and 3 gliomas (with reference to the WHO 2016 classification) were downloaded from TCGA-LGG project (The Cancer Genome Atlas; Research Network: https://www.cancer.gov/tcga, accessed on 4 October 2021) using the R package TCGAbiolinks in R statistical software (retrieved from https://www.R-project.org/; v 4.1.1) [13]. Data included in the present study were unique samples from patients with primary tumors and a survival greater than 0 days (n = 507). Patients’ characteristics are listed in Supplementary Table S1a. To compare each patient transcriptomic data with each other, the median of the ratio normalization and variance stabilizing transformation methods were applied to these data using the R package DESeq2 [14].

For data validation, transcriptomic and clinical data from grade 2 or grade 3 glioma patients were also downloaded from the CGGA database (http://www.cgga.org.cn; datasets: mRNAseq_693 and mRNAseq_325, accessed on 18 May 2022) [15,16,17,18]. Patients’ characteristics are listed in Supplementary Table S1b.

### 2.2. Selection of Differentially Expressed Genes

First of all, a differential expression analysis was performed using the R package DESeq2 [19] to identify differentially expressed genes between grade 3 and grade 2 or males and females in the TCGA database. As multiple testing was performed, we selected genes with an adjusted *p*-value of less than 1% with the Benjamini–Hochberg (BH) method.

### 2.3. Univariate Analysis

Univariate analysis was performed to test the link between patient survival (defined as the time between the date of surgery and the date of death or the date of the last follow-up) and each gene-expression level. This analysis consisted in fitting a Cox model per transcript from the R package survival. As above, the *p*-values were adjusted with the BH method with a threshold of 5%.

### 2.4. Survival Analysis

After performing a survival analysis, patients were divided into two groups of high or low (gene of interest) expression. The optimal cut-point (surv_cutpoint function, R package survminer) was obtained by using the maximally selected rank statistics, with a minimum proportion of observations per group of 25%. The log-rank test was used to compare the Kaplan–Meier survival curves between the defined high and low groups.

### 2.5. Gene Expression Correlation Analysis in the TCGA Database

From the normalized transcriptomic data of the population of interest in the TCGA database, Spearman correlation was used to identify genes with expression that was associated with that of steroid biosynthesis enzymes or hormone receptors. Genes with an absolute value of the correlation coefficient greater than 0.6 were selected.

### 2.6. Validation of Gene Expression Correlations in the CGGA Database

From the lists of genes correlated with steroid biosynthesis enzymes or hormone receptors in the TCGA database, a correlation analysis was again performed with gene expression data from the CGGA database.

### 2.7. Functional Annotation

Gene annotation was based on gene ontology and enrichment for specific biological functions was determined using FuncAssociate 2.0 program (FuncAssociate: The Gene Set Functionator; [20]) and Gene Set Enrichment Analysis (GSEA). Unsupervised GSEA was done with the whole C5 ontology gene sets from the molecular signature database (MSigDB). Upstream regulators were identified using the C3 regulatory target gene set in the GSEA database with significant FDR q-values (<0.05) [21,22].

## 3. Results

### 3.1. Grade 3 over Grade 2 Differentially Expressed Genes in Males and Females

**Figure 1:**
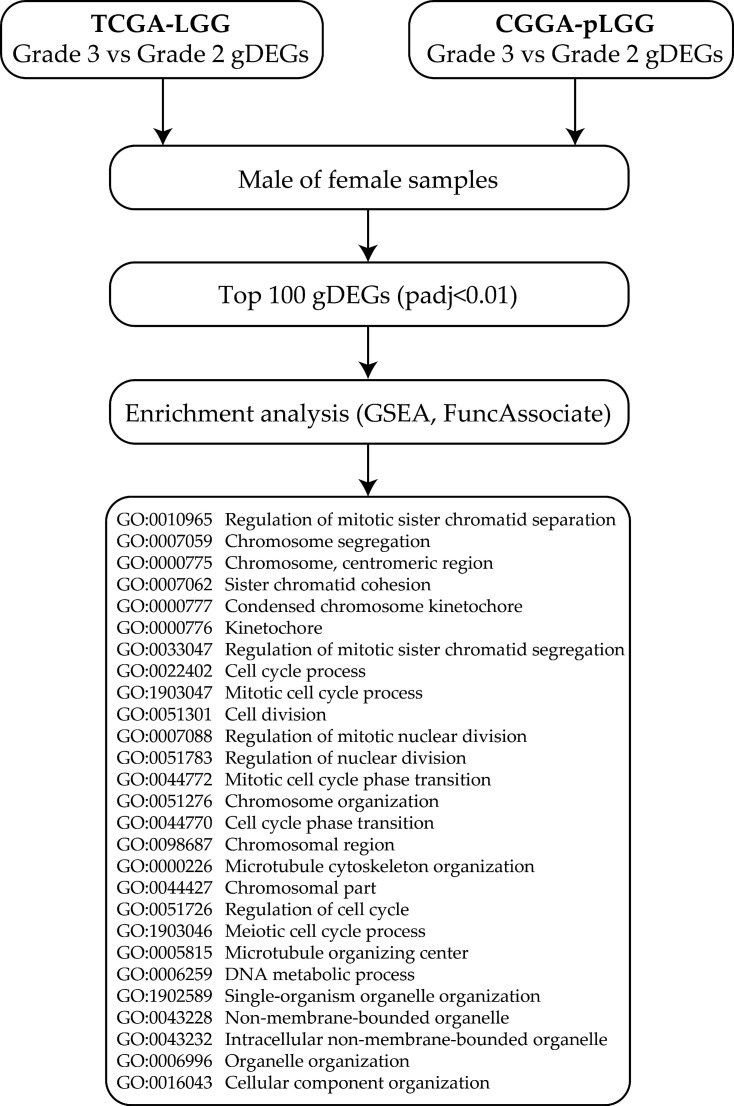
Workflow for the selection and analysis of grade 3 over grade 2 differentially expressed genes (gDEGs) from either male or female glioma samples collected from the TCGA or CGGA databases. Once identified, top 100 gDEGs were submitted to functional enrichment analysis using GSEA or FuncAssociate pipelines. GO terms common to the lists extracted from TCGA and CGGA are indicated.

The TCGA-LGG dataset contains clinical and transcriptomic data from 507 unique primary tumor samples, 215 grade 2, 236 grade 3, and 56 without histological characterization that were excluded from the present study (Supplementary Table S1a). Grade 3 over grade 2 differentially expressed genes (gDEGs) were selected from all TCGA-LGG samples (whatever the molecular status) and were stratified by gender (Figure 1). We highlighted 3042 gDEGs in males and 6496 gDEGs in females (padj < 0.01) (Supplementary Table S2). To confirm the relevance of our data, the same analysis was performed from LGG samples collected in the CGGA-pLGG database. However, the CGGA database and the TCGA database only share 22,087 transcripts in common (23,271 in CGGA database vs. 53,747 in TGCA) due to different sequencing depths. For this reason, we chose to compare gene ontologies enriched in the top 100 gDEGs. In both sexes, the top 100 gDEGs (based on minimal padj) were associated with gene ontologies related to cell division. Interestingly, most of these ontologies were also found with the top 100 gDEGs from the CGGA database, such as GO:0022402 and GO:1903047, corresponding to the cell cycle process and the mitotic cell cycle process, respectively.

Then, glioma samples were stratified by gender, IDH, and 1p/19q status: IDH wild-type (IDHwt), IDH-mutated 1p/19q non-codeleted (IDHmut), and IDH-mutated 1p/19q codeleted (IDHmut codel).

For IDHwt, the top 100 gDEGs from the TCGA database highlighted GO enrichment linked to cell division in males and females. These GO terms were also observed with females’ top 100 gDEGs from the CGGA database (Supplementary Table S3). However, there was no significant enrichment for males’ IDHwt from the CGGA database. Similarly, for IDHmut, the top 100 gDEGs from the TCGA database were linked to cell division in males and females. This finding was validated with the top 100 gDEGs from the CGGA database for females, but no GO enrichment was found for IDHmut males (Supplementary Table S3). No significant enrichment with the top100 gDEGs from the TCGA and CGGA databases for IDHmut codel males and females was found. Taken together, these data confirmed that grade 2 to grade 3 progression originated in accelerated cell division. To better characterize the role of gender in the grade 2 to grade 3 progression, we performed male-over-female DEGs analyses, from the more complete TCGA-LGG database.

### 3.2. Male-Over-Female Differentially Expressed Genes in Grade 2 or Grade 3 Gliomas Associated with Patient Overall Survival

**Figure 2:**
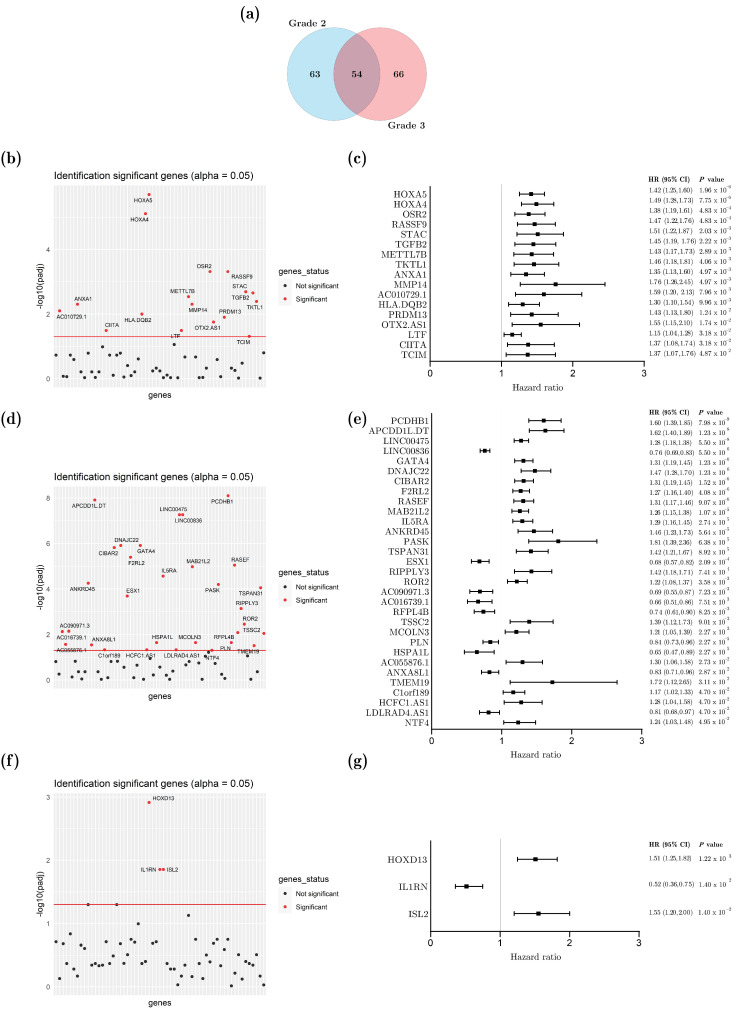
Male-over-female differentially expressed genes (DEGs) associated with patient survival in grade 2 and 3 glioma samples from TCGA. Venn diagram comparing the 117 and 120 male over-female DEGs from either grade 2 or grade 3 samples, respectively (**a**). Manhattan plots representing the association between gene-expression level and patient survival in the univariate analysis of grade 2 (**b**), grade 3 (**d**), and grade 3 IDHmut (**f**) gliomas. Red line marks significance threshold where the *p*-value adjusted by the Benjamini–Hochberg method is equal to 5%. Forest plot representing the hazard ratio (HR) and its confidence interval (CI) of genes with expression that is associated with patient survival in univariate analysis of grade 2 (**c**), grade 3 (**e**), and grade 3 IDHmut (**g**) gliomas.

Overall, 117 and 120 male-over-female DEGs were selected from the grade 2 or grade 3 TCGA-LGG samples, respectively (Supplementary Table S4). Among the 54 common DEGs identified in both grades, 30 mapped on chromosome Yp11, 8 on Xp22, 4 on Xq13, and 4 on Xq11 (Figure 2a). Since those genes were expected to be selected in a male vs female screening from any tissue, they were not included in further analyses. In contrast, a univariate analysis was performed to identify DEGs linked to patient overall survival among the 63 and 66 transcripts specifically identified from the grade 2 and grade 3 samples, respectively. In total, 17 genes with an expression level associated with patient survival were selected from grade 2 (Figure 2b,c). Gene ontology enrichment analysis indicated that six of them (Matrix Metallopeptidase *(MMP) 14*, Homeobox (*HOX)A4*, *HOXA5*, Odd-Skipped Related Transcription Factor 2 (*OSR2*), Lactotransferrin (*LTF*), and Transforming Growth Factor Beta 2 (*TGFB2*)) were involved in skeletal development [GO: 0048704; GO: 0048705; GO: 0048706; GO:0001501]. *HOXA4* and *MMP14* might be regulated by the EN1 (Engrailed 1) upstream transcription factor, together with Ras Association Domain Family Member 9 (RASSF9) or PR/SET Domain 13 (*PRDM13*), respectively. Then, 31 DEGs were associated to survival in grade 3 tumors (Figure 2d,e and Supplementary Table S4), but this list displayed no enrichment in GO terms or upstream transcription factor. Male-over-female DEGs were further selected and analyzed depending on the three glioma molecular subtypes. No subtype-specific DEGs linked to patient survival could be identified from grade 2 tumors, due to the small number of patients who died during the study (31/215 = 14.4%), with respect to the large number of living individuals excluded from the study (85.6%). From grade 3 tumors, 22, 59, and 32 DEGs were selected from IDHwt, IDHmut, and IDHmut codel tumors, respectively (Supplementary Table S5).

Based on a univariate analysis, only three DEGs identified from the IDHmut samples were significantly (padj < 0.05) associated with patient survival: ISL LIM Homeobox 2 (*ISL*2), Interleukin 1 Receptor Antagonist (*IL1RN*), and *HOXD*13 (Figure 2f,g).

### 3.3. Expression of Aromatase and Steroid Receptors in Grade 3 Tumors Associated with Patient Overall Survival Depending on Gender and Tumor Type

**Figure 3:**
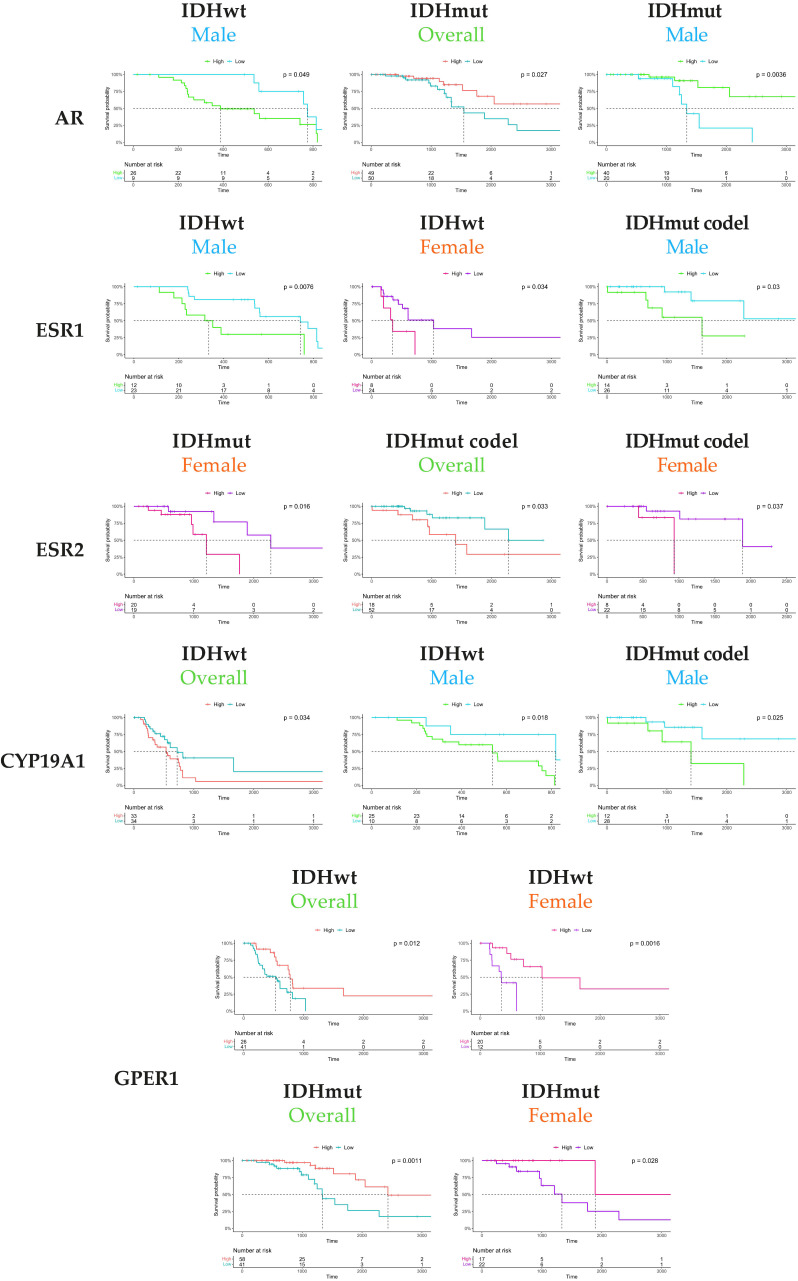
Kaplan–Meier survival curves of IDHwt, IDHmut, or IDHmut codel grade 3 patients (overall population, males, females) according to the expression of *AR*, *ESR*1, *ESR*2, *CYP*19*A1*, and *GPER*1. The curves were selected when corresponding gene expression was significantly associated to patient survival (*p* < 0.05). The optimal cut-point was obtained by using the maximally selected rank statistics, with a minimum proportion of observations per group of 25%.

Since only a few genes associated with survival were identified in the previous screening, we assumed that the clinical differences between males and females observed in the natural history of the tumors might originate from hormone production and/or signaling. From the TCGA-LGG RNAseq and clinical data, we performed a supervised analysis of the potential correlation between key steroid biosynthesis enzyme (*CYP*19*A*1 encoding aromatase) or receptor (*AR*, *ESR*1, *ESR*2, *GPER*1, *PGR*) expression and grade 3 patient survival, depending on the glioma type and patient gender (Figure 3, Table 1). *PGR* expression was not linked to survival, whatever the gender or type and was, therefore, not selected for further analysis.

A low *AR* expression was associated with a longer survival in IDHwt males, whereas a high *AR* expression was of good prognosis in IDHmut grade 3 patients overall and in the male population. A low *ESR*1 and *ESR*2, as well as a low *CYP*19*A*1 expression, were associated with good prognosis, suggesting that a low level of active estrogens might dampen anaplastic transformation. Low *ESR*1 expression was of good prognosis in IDHwt tumors both in males and females, though low *ESR*1 expression was of good prognosis in IDHmut codel tumors only in males. Low *ESR*2 expression was related to longer survival only in females in IDHmut/codel, while low *CYP*19*A*1 expression was associated with survival in IDHwt and IDHmut codel in males. Conversely, a high *GPER*1 expression was linked to a better survival in IDHwt and IDHmut types overall. These observations were significant in both types female tumors. Taken together, these results indicated that estrogen nuclear receptor signaling greatly differed from membrane-initiated ones, whereas both appeared to contribute to patient survival.

### 3.4. Genes Correlated with Aromatase and Steroid Receptor Expression in the Whole Population, Males, and Females, Stratified by Tumor Type

We then wanted to gain additional insight into the functionality of aromatase and steroid receptors in each grade 3 type where their expression was correlated with patient survival. We performed a co-expression correlation analysis (Supplemental Table S6 |rho| > 0.6). Of note, all the correlated genes cited in Table 1 were validated with the CGGA database.

We identified 11 genes positively correlated with *AR* expression in IDHwt in males. These genes were associated with a GO term related to the amino acid betaine biosynthetic process, as assessed by the expression of Gamma-Butyrobetaine Hydroxylase 1 (*BBOX1*). No significant enrichment was found for *AR* correlated genes in the IDHmut type. In total, 44 genes were positively correlated with *ESR1* in IDHwt, whereas no *ESR1* correlated gene was highlighted in males. These 44 genes were related to adaptative immune response, as supported by the presence of the T-Cell Surface *CD2* surface antigen or the T-Cell Surface Glycoprotein CD3 Gamma Chain (*CD3G*). No specific functions were enriched in the 29- and 9-gene signatures correlated with *ESR*2 expression in IDHmut and IDHmut codel tumors, respectively. We highlighted a 23-gene signature positively correlated with *CYP*19*A*1 expression in IDHwt tumors in the overall population. These genes were linked to biological adhesion and immune response, including the Fc Epsilon Receptor Ig (*FCER*1*G*) or the adhesion molecule Sialic Acid Binding Ig Like Lectin 9 (*SIGLEC*9) and the adhesion molecule Sialic Acid Binding Ig Like Lectin 7 (*SIGLEC*7). Most of these genes were also corelated to *CYP*19*A*1 in males. Interestingly, the 66-gene signature in IDHmut codel tumors was also related to innate immune response such as the Complement Component 1, R Subcomponent (*C*1*R*). The 50 genes correlated with *GPER1* expression in IDHwt tumors in the whole population were involved in vasculature development through expression of cell-surface receptors such as Activin A Receptor Like Type 1 (*ACVRL*1) or the vascular endothelial growth factor receptor (*FLT*4). These genes were also correlated with *GPER*1 in females. Finally, the *GPER1* negatively correlated genes of the 191 gene-signature in IDHmut females were associated with nucleic acid process, as supported by the Transcription Factor Dp-2 (*TFDP*2) or numerous Zinc Finger Protein (*ZNF*) members.

## 4. Discussion

Low-grade and anaplastic gliomas are considered as relatively rare tumors, with an incidence in the USA estimated at 0.51 and 0.25 per 100,000 per year for astrocytoma and oligodendroglioma, respectively [23]. Consequently, large cohorts remain barely available, except from public databases. In the present study, we mined the TCGA-LGG and CGGA databases to gain insight into the molecular basis of gender differences in the glioma natural history. Since glioma classification is constantly being updated since 2016, we stratified the samples not only according to the histological grade but also based on the molecular criteria such as IDHwt/mut or 1p/19q codeletion status.

In clinics, anaplastic transformation with accelerated tumor growth and aggressivity characterizes grade 2 to 3 transition. Transcriptomic data from either TCGA or CGGA datasets supported these observations, since most genes affected were involved in cell cycle, cell division, and chromosome segregation in males and females. However, IDHwt male tumors displayed a high level of gene expression heterogeneity, and no molecular pathway characterized grade 2 to grade 3 transformation. A more refined classification seems necessary for this group to ensure the robustness of the clinical prognosis and to adapt patient care.

Then, we focused on either grade 2 or grade 3 tumors and identified male-over-female DEG. As previously described, such DEG screening trivially identified many X- or Y-linked genes because of their sexually dimorphic expression. In addition, approximately 70% of the DEGs we identified have been previously found in an analysis mixing low-grade and anaplastic grade 3 gliomas [5]. However, the grade stratification in our study allowed the identification of new grade-2-specific genes that were involved in skeletal morphogenesis (*MMP*14, *HOXA*4, *HOXA*5, *OSR*2, *LTF*, *TGFB*2). *HOX*4*A* and *HOX*5*A* homeobox genes belonged to clusters that encode transcription factors required for normal development, which are repressed in healthy adult tissues but often deregulated in brain tumors [24]. Recently, Le Boiteux et al. (2021) demonstrated that HOX clusters reactivated in aggressive IDHwt gliomas following loss of H3K27me3 and hypermethylation escape [25]. HOXA5 was also involved in cell proliferation and apoptosis control and identified as a prognostic-related biomarker in GBM [26]. Moreover, the gene enrichment analysis identified *EN*1 as an upstream transcription factor for grade-2-specific DEGs. The expression of this member of homeobox-containing transcription factors, was previously shown to correlate with shorter survival in low-grade glioma patients, irrespective of IDH status or 1p/19q codeletion [27].

Further, two of the three DEGs associated with patient survival in type stratified grade 3 tumors were homeobox-related genes. A low expression of *HOXD*13 correlated to better survival in IDHmut grade 3 patients, which supported previous data indicating that a hypermethylation of *HOXD*13 was observed in IDH-mutated GBM-patient long-term survivors, compared to short-term survivors [28]. Zhang et al. (2020) demonstrated that miR-7156-3p modulated *HOXD13* expression in GBM and thus, lowered GBM progression by regulating tumor cell stemness [29]. A low expression of the ISL2 appeared to be of good prognosis, regardless of the type of grade 3 tumor. Overexpression of this transcription factor was shown to participate in a feedback loop with miR342-3p, to promote angiogenesis in GBM and control cell proliferation and malignant transformation in oligodendroglioma [30].

Taken together, the results from the present study further supported the potential role of HOX genes expression as a prognosis factor in low-grade and anaplastic gliomas, together with upstream regulators. This suggested that this disease onset might be related to the epigenetic alteration of developmental patterning, which promoted the tumor anaplastic feature through stem cell maintenance and angiogenesis. Since these HOX-related genes were selected as male-over-female DEGs, we wondered which of their common drivers could also be different between males and females. The hormonal hypothesis appeared relevant, since HOX gene expression was controlled by steroid hormones, namely estrogens and progesterone, during normal reproductive-tract development and in adult tissue, such as endometrium during the menstrual cycle. HOX clusters were also dysregulated in hormone-sensitive diseases such as endometriosis, breast or prostate cancers (for review [31]). Moreover, strong estrogen-mimicking compounds such as diethylstilbestrol (DES) or bisphenol A (BPA) might impair correct HOX patterning in sexually dimorphic organs through genetic and epigenetic alterations and lead to functional abnormalities [32,33].

Consequently, we addressed the potential association between grade 3 glioma patient survival and steroid biosynthesis enzyme or receptor expression. We showed that in addition to a stratification according to the defined glioma subtypes, a gender-stratification allowed to better define the role of steroid signaling in grade 3 gliomas and the prognostic potential of these gene expressions. Indeed, AR and aromatase were associated with patient survival in males, whereas estrogen signaling (involving ERα, Erβ, and GPER) seemed to play a major role in females. In both cases, these observations were tumor-subtype specific.

Consistent with previous findings showing that AR had pro-tumor properties in GBM tumor cells, we found in grade 3 gliomas that a low *AR* expression associated with better survival and one-carbon metabolism, especially in male IDHwt tumors. This suggested that AR-dependent signaling could influence the epigenetic feature of these tumors by altering the gene methylation level [6,34]. Surprisingly, a high level of *AR* was of good prognosis in IDHmut tumors, especially in males, but no associated function could be inferred from gene-correlation analysis. Conflicting results previously indicated that *AR* expression was either higher or lower in GBM than in low-grade gliomas (reviewed in [6]). This underlined the need to cautiously consider *AR* expression as a predictive marker for survival, according to tumor subtype.

*PGR* never associated with survival, while several studies highlighted an accelerated growth and anaplastic transformation of low-grade tumors during pregnancy, a unique hormonal period characterized by huge levels of progesterone and estrogens [10].

Low canonical estrogen signaling, mediated by estrogens (produced by *CYP*19*A*1 encoded protein) and the ERα nuclear receptor, appeared to be linked to immune response and the adhesive interactions between immune cells in IDHwt and IDHmut codel grade 3 tumors. These findings support the role of immunity in the prognosis of anaplastic glioma. Indeed, an immune-response signature was found in the IDHwt high-risk group and was correlated with a poor prognosis for LGG patients [35]. Recently, Song et al. identified a 120-gene immune infiltration signature differentially expressed in low-grade glioma, compared with the normal brain, and selected a 20-gene immune infiltration signature that could predict the survival of the patients. Among them, *SPP*1 and genes of the SIGLEC family, encoding Ig, such as lectins, were found correlated with *CYP*19*A*1 in IDHwt and IDHmut 1p/19q codel tumors and associated with a poor prognosis [36]. Our study is the first one showing the link between the molecular status, aromatase, nuclear estrogen receptors’ expression, and immune response. ESR2 expression was found to be of poor prognosis in IDHmut (1p/19q codel or not) female tumors, but no correlated genes or functions were identified. Recently, Liu and coworkers observed a differential deregulation of ERβ isoforms in GBM and demonstrated that ERβ1 has a tumor-suppression function, while ERβ5 promoted oncogenic function [37]. Further work should give more insight on the prognosis value of each ERβ isoforms in female IDHmut low-grade gliomas.

Conversely to the low level of nuclear receptors, a high expression level of membrane estrogen receptor *GPER1* correlated with better survival in both IDHwt and IDHmut female patients, in connection with vasculature development and the RNA metabolic process, respectively. A high *GPER1* level associated with a high expression of key genes involved in angiogenesis such as *PDGFRB*, which enhanced glioma cell migration and was proposed together with EGFR as a target for therapeutic agents [38,39]. Ambhore et al. demonstrated that ERβ agonists, but not the GPER*-*agonist G-1, significantly decreased PDGF production and the proliferation of airway smooth muscle cells through non-genomic signaling [40]. Recently, Hirtz et al. (2021) showed that *GPER*1 expression was also of good prognosis in the GBM tumors listed in the TCGA-GBM database and GBM cell lines, although G-1 triggered GPER-independent cell cycle arrest [41]. The 17β-estradiol and G-1 affinity and selectivity for GPER is increasingly being questioned [42]. In grade 3 tumors, we can, therefore, hypothesize that GPER might either participate in estrogen non-genomic signaling or respond to a still unknown stimulus to inhibit angiogenesis and improve patient survival. Therefore, the detailed gene network that relates GPER signaling to angiogenesis modulation, anaplastic progression, and survival appeared of great interest in the perspective of new targeted therapies. Indeed, the *GPER*1 expression level appeared to be of high prognostic value, especially for female patients. In IDHwt and IDHmut female tumors, female-specific genes negatively corelated with *GPER*1 were mainly regulated by miR548, which has been associated with impaired proliferation in various cancers [43], including glioma [44] and hormone-sensitive tumors such as prostate cancer [45] and breast cancer [43,46]. In IDHmut tumors, female-specific genes positively associated to *GPER1* were mainly regulated by upstream factors such as HMG1, which mediates GBM regression and promotes angiogenesis [47], and ERRα, with expression that increases with glioma grade [48]. In GBM, ERRα promotes Wnt5 expression and poor prognosis but might be targeted by several natural or xenobiotic compounds [49].

In conclusion, our results confirm the relevance of the gender-related mechanisms involved in low-grade and anaplastic gliomas by highlighting steroid-related genes and specific signaling (such as immune or angiogenic pathways) as new prognostic markers in diffuse gliomas. The results obtained in this retrospective study could not take into account all the new molecular markers allowing the classification of gliomas. Since central nervous system tumor classification is being constantly updated with new genetic alterations, their links with steroid biosynthesis/signaling will be explored as the databases are updated to open new perspectives for targeted therapies.

## Figures and Tables

**Table 1:** Type-specific correlation between key steroid biosynthesis enzyme or hormone receptor expression and patient survival. For each type (IDHwt, IDHmut, IDHmut codel) the correlation between the expression of *AR*, *ESR*1, *ESR*2, *CYP*19*A*1, or *GPER*1 with survival was assessed. The expression level (low or high) corresponding to good prognosis was indicated. GO enrichment analysis of the genes correlated with *AR*, *ESR*1, *ESR*2, *CYP*19*A*1, or *GPER*1 was performed: number of correlated genes, top significant GO terms, enrichment pvalue, and FDRqvalue as well as genes associated with enrichment GO terms, as identified from both TCGA and CGGA databases (based on absolute rho value), were indicated.

				Correlated Genes (IrhoI > 0.6)					
		Gender	Good Prognosis	Number	Top GO		pval	FDR qval	Genes Associated with Enriched GO (TCGA and CGGA)
**AR**	IDH wt	Male	Low	11	GO:0006578	amino-acid betaine biosynthetic process	8.11.10^−7^	8.44.10^−3^	BBOX1
	IDH mut	All	High	3	-	-	-	-	-
		Male	High	19	-	-	-	-	-
**ESR1**	IDH wt	Male	Low	0			-	-	-
		Female	Low	44	GO:0006955	immune response	1.34.10^−26^	1.4.10^−22^	CD3G, EOMES, SLAMF1, SLAMF7, CCR4, CD3E, GZMA, CST7, LY9, CD96, FCGR2B, IL2RG, SLAMF6, FASLG, SKAP1
					GO:0042110	T cell activation	1.10^−18^	1.74.10^−15^	CD3G, EOMES, CD2, CD3E, LCK
	IDH mut codel	Male	Low	4	-	-	-	-	-
**ESR2**	IDH mut	Female	Low	29	-	-	-	-	-
	IDH mut codel	All	Low	9	-	-	-	-	-
		Female	Low	0	-	-	-	-	-
**CYP19A1**	IDHwt	All	Low	23	GO:0022610	Biological adhesion	6.7.10^−8^	5.63.10^−4^	RAC2, PLAUR, SIGLEC9, SIGLEC7, SPP1, FRMT3, S100A11
					GO:0002274	Myeloid leukocyte activation	1.84.10^−7^	5.63.10^−4^	RAC2, FCER1G, HMOX1
		Male	Low	22	GO:0022610	Biological adhesion	3.27.10^−6^	3.4.10^−2^	RAC2, PLAUR, SIGLEC9, SIGLEC7, CSTA, IL4I1, SPP1
	IDH mut codel	Male	Low	66	GO:0045087	Innate immune response	2.42.10^−19^	4.19.10^−16^	C1R, APOL1, HLA-E
**GPER1**	IDH wt	All	High	50	GO:0001944	vasculature development	3.9.10^−19^	4.06.10^−15^	FLT4, TIE1, ACVRL1, CLEC14A, PDGFRB, ADGRA2, ROBO4, NOTCH4, ANPEP, CDH5, ECM1, RASIP1, TMEM204, ARHGEF15
		Female	High	85	GO:0001944	Vasculature development	3.53.10^−15^	3.67.10^−11^	FLT4, NOTCH4, ACVR1L, CLEC14A, RASIP1, EGFL7, ROBO4, CLDN5
	IDH mut	All	High	1	-	-	-	-	-
		Female	High	191	GO:0016070	RNA metabolic process	5.32.10^−10^	1.47.10^−6^	ZNF333, ZNF254, TFDP2, MGA, SETD5, ZFP69, SRSF11, PNN, HNRNPH1, TRIT1, ZNF326

## Data Availability

The results published here are based upon data generated by the TCGA Research Network at https://www.cancer.gov/tcga and the CGGA database at http://www.cgga.org.cn. Data from the TCGA-LGG dataset are available at https://portal.gdc.cancer.gov/projects/TCGA-LGG and were accessed on 4 October 2021. Data from the CGGA database are available at http://www.cgga.org.cn/download.jsp#mRNAseq_693 for the mRNAseq_693 dataset and at http://www.cgga.org.cn/download.jsp#mRNAseq_325 for the mRNAseq_325 dataset. Data were accessed on 18 May 2022.

## Supplementary Materials

The following supporting information can be downloaded at: https://www.mdpi.com/article/10.3390/cancers14174114/s1. Table S1: Patient characteristics of TCGA-LGG (a) and CGGA-LGG databases. IDH, isocitrate dehydrogenase; NA: not applicable. Table S2: List of grade 3 over grade 2 differentially expressed genes (gDEGs) from male (a) and female (b) LGG samples collected from the TCGA database. Table S3: GO terms significantly enriched from grade 3 over grade 2 DEGs lists in IDHwt or IDHmut tumors from the TCGA and CGGA databases. Table S4: Lists of male over female DEGs in grade 2 and/or grade 3 tumors. Table S5: Lists of male over female DEGs in IDHwt, IDHmut, and IDHmut codel grade 3 tumors. Table S6: Lists of genes whose expression significantly associated with the indicated receptor or aromatase in IDHwt, IDHmut, and IDHmut codel grade 3 tumors stratified by gender. Positive correlations (rho > 0.6) are shown in red and negative correlations (rho < −0.6) are shown in green.

## References

[1] Louis D.N., Perry A., Wesseling P., Brat D.J., Cree I.A., Figarella-Branger D., Hawkins C., Ng H.K., Pfister S.M., Reifenberger G. (2021). The 2021 WHO Classification of Tumors of the Central Nervous System: A Summary. Neuro-Oncology.

[2] Obara T., Blonski M., Brzenczek C., Mézières S., Gaudeau Y., Pouget C., Gauchotte G., Verger A., Vogin G., Moureaux J.-M. (2020). Adult Diffuse Low-Grade Gliomas: 35-Year Experience at the Nancy France Neurooncology Unit. Front. Oncol..

[3] Brown T.J., Bota D.A., van Den Bent M.J., Brown P.D., Maher E., Aregawi D., Liau L.M., Buckner J.C., Weller M., Berger M.S. (2019). Management of Low-Grade Glioma: A Systematic Review and Meta-Analysis. Neuro-Oncol. Pract..

[4] Ostrom Q.T., Gittleman H., Fulop J., Liu M., Blanda R., Kromer C., Wolinsky Y., Kruchko C., Barnholtz-Sloan J.S. (2015). CBTRUS Statistical Report: Primary Brain and Central Nervous System Tumors Diagnosed in the United States in 2008–2012. Neuro Oncol..

[5] Khan M.T., Prajapati B., Lakhina S., Sharma M., Prajapati S., Chosdol K., Sinha S. (2021). Identification of Gender-Specific Molecular Differences in Glioblastoma (GBM) and Low-Grade Glioma (LGG) by the Analysis of Large Transcriptomic and Epigenomic Datasets. Front. Oncol..

[6] Hirtz A., Rech F., Dubois-Pot-Schneider H., Dumond H. (2020). Astrocytoma: A Hormone-Sensitive Tumor?. Int. J. Mol. Sci..

[7] Yu X., Jiang Y., Wei W., Cong P., Ding Y., Xiang L., Wu K. (2015). Androgen Receptor Signaling Regulates Growth of Glioblastoma Multiforme in Men. Tumour Biol..

[8] Weidenfeld J., Schiller H. (1984). Metabolism of Steroids by Human Brain Tumors. Clin. Neuropharmacol..

[9] Pallud J., Mandonnet E., Deroulers C., Fontaine D., Badoual M., Capelle L., Guillet-May F., Page P., Peruzzi P., Jouanneau E. (2010). Pregnancy Increases the Growth Rates of World Health Organization Grade II Gliomas. Ann. Neurol..

[10] Peeters S., Pagès M., Gauchotte G., Miquel C., Cartalat-Carel S., Guillamo J.-S., Capelle L., Delattre J.-Y., Beauchesne P., Debouverie M. (2018). Interactions between Glioma and Pregnancy: Insight from a 52-Case Multicenter Series. J. Neurosurg..

[11] Tavares C., Gomes-Braga F., Costa-Silva D., Escórcio-Dourado C., Borges U., Conde-Junior A., Barros-Oliveira M., Sousa E., Barros L., Martins L. (2016). Expression of Estrogen and Progesterone Receptors in Astrocytomas: A Literature Review. Clinics.

[12] Dueñas Jiménez J.M., Candanedo Arellano A., Santerre A., Orozco Suárez S., Sandoval Sánchez H., Feria Romero I., López-Elizalde R., Alonso Venegas M., Netel B., de la Torre Valdovinos B. (2014). Aromatase and Estrogen Receptor Alpha MRNA Expression as Prognostic Biomarkers in Patients with Astrocytomas. J. Neurooncol..

[13] Colaprico A., Silva T.C., Olsen C., Garofano L., Cava C., Garolini D., Sabedot T.S., Malta T.M., Pagnotta S.M., Castiglioni I. (2016). TCGAbiolinks: An R/Bioconductor Package for Integrative Analysis of TCGA Data. Nucleic Acids Res..

[14] Love M.I., Huber W., Anders S. (2014). Moderated Estimation of Fold Change and Dispersion for RNA-Seq Data with DESeq2. Genome Biol..

[15] Zhao Z., Zhang K.-N., Wang Q., Li G., Zeng F., Zhang Y., Wu F., Chai R., Wang Z., Zhang C. (2021). Chinese Glioma Genome Atlas (CGGA): A Comprehensive Resource with Functional Genomic Data from Chinese Glioma Patients. Genom. Proteom. Bioinform..

[16] Liu X., Li Y., Qian Z., Sun Z., Xu K., Wang K., Liu S., Fan X., Li S., Zhang Z. (2018). A Radiomic Signature as a Non-Invasive Predictor of Progression-Free Survival in Patients with Lower-Grade Gliomas. Neuroimage Clin..

[17] Zhao Z., Meng F., Wang W., Wang Z., Zhang C., Jiang T. (2017). Comprehensive RNA-Seq Transcriptomic Profiling in the Malignant Progression of Gliomas. Sci. Data.

[18] Wang Y., Qian T., You G., Peng X., Chen C., You Y., Yao K., Wu C., Ma J., Sha Z. (2015). Localizing Seizure-Susceptible Brain Regions Associated with Low-Grade Gliomas Using Voxel-Based Lesion-Symptom Mapping. Neuro Oncol..

[19] Goel M.K., Khanna P., Kishore J. (2010). Understanding Survival Analysis: Kaplan-Meier Estimate. Int. J. Ayurveda Res..

[20] Berriz G.F., Beaver J.E., Cenik C., Tasan M., Roth F.P. (2009). Next Generation Software for Functional Trend Analysis. Bioinformatics.

[21] Liberzon A., Birger C., Thorvaldsdóttir H., Ghandi M., Mesirov J.P., Tamayo P. (2015). The Molecular Signatures Database (MSigDB) Hallmark Gene Set Collection. Cell Syst..

[22] Subramanian A., Tamayo P., Mootha V.K., Mukherjee S., Ebert B.L., Gillette M.A., Paulovich A., Pomeroy S.L., Golub T.R., Lander E.S. (2005). Gene Set Enrichment Analysis: A Knowledge-Based Approach for Interpreting Genome-Wide Expression Profiles. Proc. Natl. Acad. Sci. USA.

[23] Youssef G., Miller J.J. (2020). Lower Grade Gliomas. Curr. Neurol. Neurosci. Rep..

[24] Gonçalves C.S., Le Boiteux E., Arnaud P., Costa B.M. (2020). HOX Gene Cluster (de)Regulation in Brain: From Neurodevelopment to Malignant Glial Tumours. Cell. Mol. Life Sci..

[25] Le Boiteux E., Court F., Guichet P., Vaurs-Barrière C., Vaillant I., Chautard E., Verrelle P., Costa B.M., Karayan-Tapon L., Fogli A. (2021). Widespread Overexpression from the Four DNA Hypermethylated HOX Clusters in Aggressive (*IDH* Wt) Glioma Is Associated with H3K27me3 Depletion and Alternative Promoter Usage. Mol. Oncol..

[26] Ding F., Chen P., Bie P., Piao W., Cheng Q. (2021). HOXA5 Is Recognized as a Prognostic-Related Biomarker and Promotes Glioma Progression Through Affecting Cell Cycle. Front. Oncol..

[27] Zhu J., Zhang Y.-Q. (2019). Engrailed 1 Overexpression as a Potential Prognostic Marker in Lower Grade Glioma. PeerJ.

[28] Shinawi T., Hill V.K., Krex D., Schackert G., Gentle D., Morris M.R., Wei W., Cruickshank G., Maher E.R., Latif F. (2013). DNA Methylation Profiles of Long- and Short-Term Glioblastoma Survivors. Epigenetics.

[29] Zhang J., Deng M., Tong H., Xue W., Guo Y., Wang J., Chen L., Wang S. (2020). A Novel MiR-7156-3p-HOXD13 Axis Modulates Glioma Progression by Regulating Tumor Cell Stemness. Int. J. Biol. Sci..

[30] Jiang Y., Zhou J., Zhao J., Zhang H., Li L., Li H., Chen L., Hu J., Zheng W., Jing Z. (2020). The U2AF2 /CircRNA ARF1/MiR-342–3p/ISL2 Feedback Loop Regulates Angiogenesis in Glioma Stem Cells. J. Exp. Clin. Cancer Res..

[31] Daftary G.S., Taylor H.S. (2006). Endocrine Regulation of HOX Genes. Endocr. Rev..

[32] Akbas G.E., Song J., Taylor H.S. (2004). A HOXA10 Estrogen Response Element (ERE) Is Differentially Regulated by 17 Beta-Estradiol and Diethylstilbestrol (DES). J. Mol. Biol..

[33] Preciados M., Yoo C., Roy D. (2016). Estrogenic Endocrine Disrupting Chemicals Influencing NRF1 Regulated Gene Networks in the Development of Complex Human Brain Diseases. Int. J. Mol. Sci..

[34] Sapienza C., Issa J.-P. (2016). Diet, Nutrition, and Cancer Epigenetics. Annu. Rev. Nutr..

[35] Han T., Zuo Z., Qu M., Zhou Y., Li Q., Wang H. (2021). Comprehensive Analysis of Inflammatory Response-Related Genes, and Prognosis and Immune Infiltration in Patients With Low-Grade Glioma. Front. Pharmacol..

[36] Song L.-R., Weng J.-C., Li C.-B., Huo X.-L., Li H., Hao S.-Y., Wu Z., Wang L., Li D., Zhang J.-T. (2020). Prognostic and Predictive Value of an Immune Infiltration Signature in Diffuse Lower-Grade Gliomas. JCI Insight.

[37] Liu J., Sareddy G.R., Zhou M., Viswanadhapalli S., Li X., Lai Z., Tekmal R.R., Brenner A., Vadlamudi R.K. (2018). Differential Effects of Estrogen Receptor β Isoforms on Glioblastoma Progression. Cancer Res..

[38] Wallmann T., Zhang X.-M., Wallerius M., Bolin S., Joly A.-L., Sobocki C., Leiss L., Jiang Y., Bergh J., Holland E.C. (2018). Microglia Induce PDGFRB Expression in Glioma Cells to Enhance Their Migratory Capacity. iScience.

[39] Xu G., Li J.Y. (2016). Differential Expression of PDGFRB and EGFR in Microvascular Proliferation in Glioblastoma. Tumour Biol..

[40] Ambhore N.S., Katragadda R., Raju Kalidhindi R.S., Thompson M.A., Pabelick C.M., Prakash Y.S., Sathish V. (2018). Estrogen Receptor Beta Signaling Inhibits PDGF Induced Human Airway Smooth Muscle Proliferation. Mol. Cell. Endocrinol..

[41] Hirtz A., Lebourdais N., Rech F., Bailly Y., Vaginay A., Smaïl-Tabbone M., Dubois-Pot-Schneider H., Dumond H. (2021). GPER Agonist G-1 Disrupts Tubulin Dynamics and Potentiates Temozolomide to Impair Glioblastoma Cell Proliferation. Cells.

[42] Tutzauer J., Gonzalez de Valdivia E., Swärd K., Alexandrakis Eilard I., Broselid S., Kahn R., Olde B., Leeb-Lundberg L.M.F. (2021). Ligand-Independent G Protein–Coupled Estrogen Receptor/G Protein–Coupled Receptor 30 Activity: Lack of Receptor-Dependent Effects of G-1 and 17 *β*-Estradiol. Mol. Pharmacol..

[43] Guo X., Lee S., Cao P. (2019). The Inhibitive Effect of Sh-HIF1A-AS2 on the Proliferation, Invasion, and Pathological Damage of Breast Cancer via Targeting MiR-548c-3p through Regulating HIF-1α/VEGF Pathway in Vitro and Vivo. OncoTargets Ther..

[44] Lu J., Zhang M., Yang X., Cui T., Dai J. (2017). MicroRNA-548c-3p Inhibits T98G Glioma Cell Proliferation and Migration by Downregulating c-Myb. Oncol. Lett..

[45] Saffari M., Ghaderian S.M.H., Omrani M.D., Afsharpad M., Shankaie K., Samadaian N. (2018). The Association of MiR-Let 7b and MiR-548 with PTEN in Prostate Cancer. Urol. J..

[46] Tormo E., Pineda B., Serna E., Guijarro A., Ribas G., Fores J., Chirivella E., Climent J., Lluch A., Eroles P. (2015). MicroRNA Profile in Response to Doxorubicin Treatment in Breast Cancer. J. Cell Biochem..

[47] Seidu R.A., Wu M., Su Z., Xu H. (2017). Paradoxical Role of High Mobility Group Box 1 in Glioma: A Suppressor or a Promoter?. Oncol. Rev..

[48] Lynch C., Zhao J., Sakamuru S., Zhang L., Huang R., Witt K., Merrick B., Teng C., Xia M. (2019). Identification of Compounds That Inhibit Estrogen-Related Receptor Alpha Signaling Using High-Throughput Screening Assays. Molecules.

[49] Zhang L., Zhu Y., Cheng H., Zhang J., Zhu Y., Chen H., Chen L., Qi H., Ren G., Tang J. (2019). The Increased Expression of Estrogen-Related Receptor α Correlates with Wnt5a and Poor Prognosis in Patients with Glioma. Mol. Cancer Ther..

