# Supplementary figures and images for: Identification of Gender- and Subtype-Specific Gene Expression Associated with Patient Survival in Low-Grade and Anaplastic Glioma in Connection with Steroid Signaling
Source: Cancers (Basel). 2022 Aug 25;14(17):4114. doi: 10.3390/cancers14174114 (PMC9454517; doi:10.3390/cancers14174114)

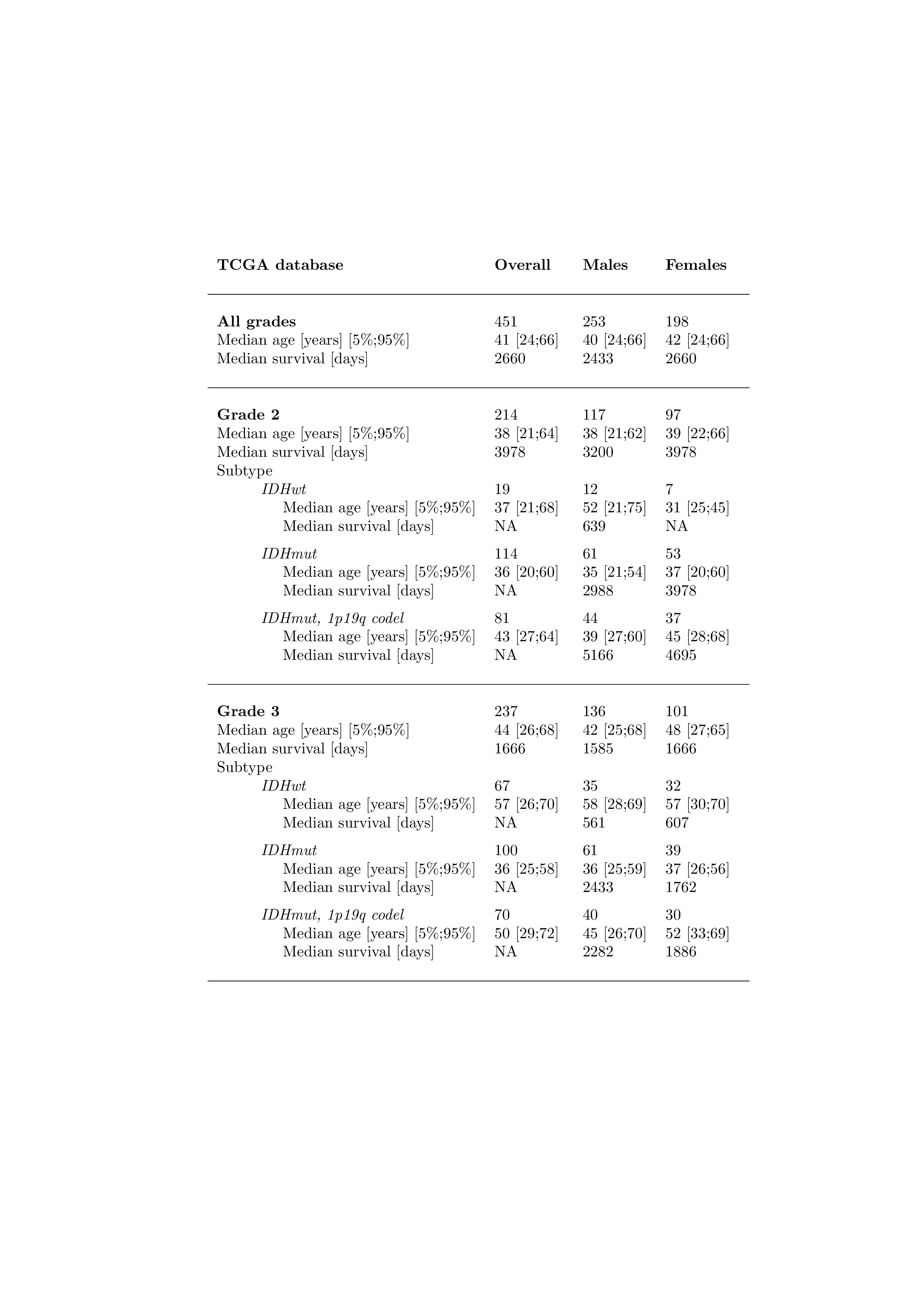

Supplement: Supplementary file 1 [file cancers-14-04114-s001.zip › Supplementary Table S1a.png]

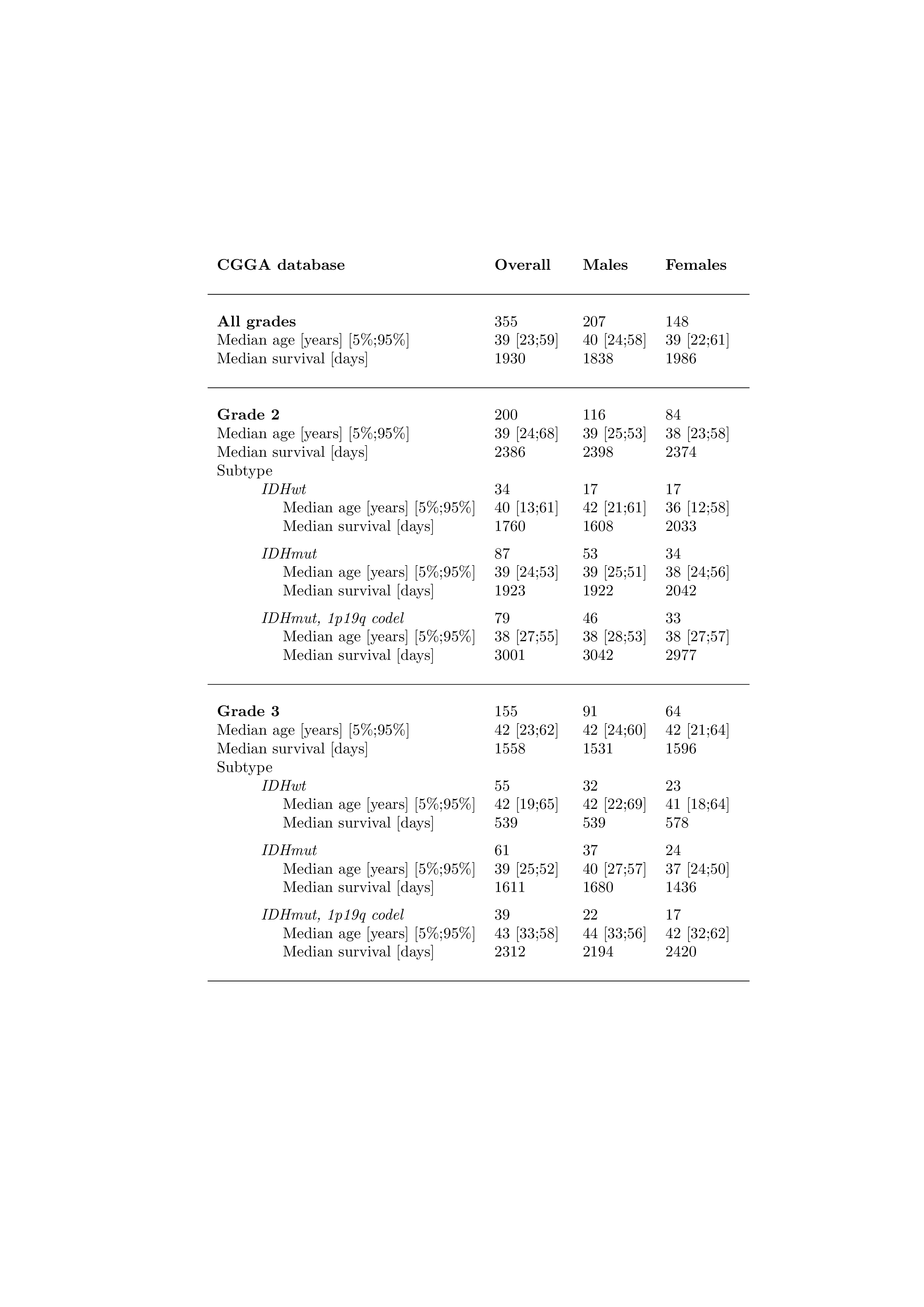

Supplement: Supplementary file 1 [file cancers-14-04114-s001.zip › Supplementary Table S1b.png]

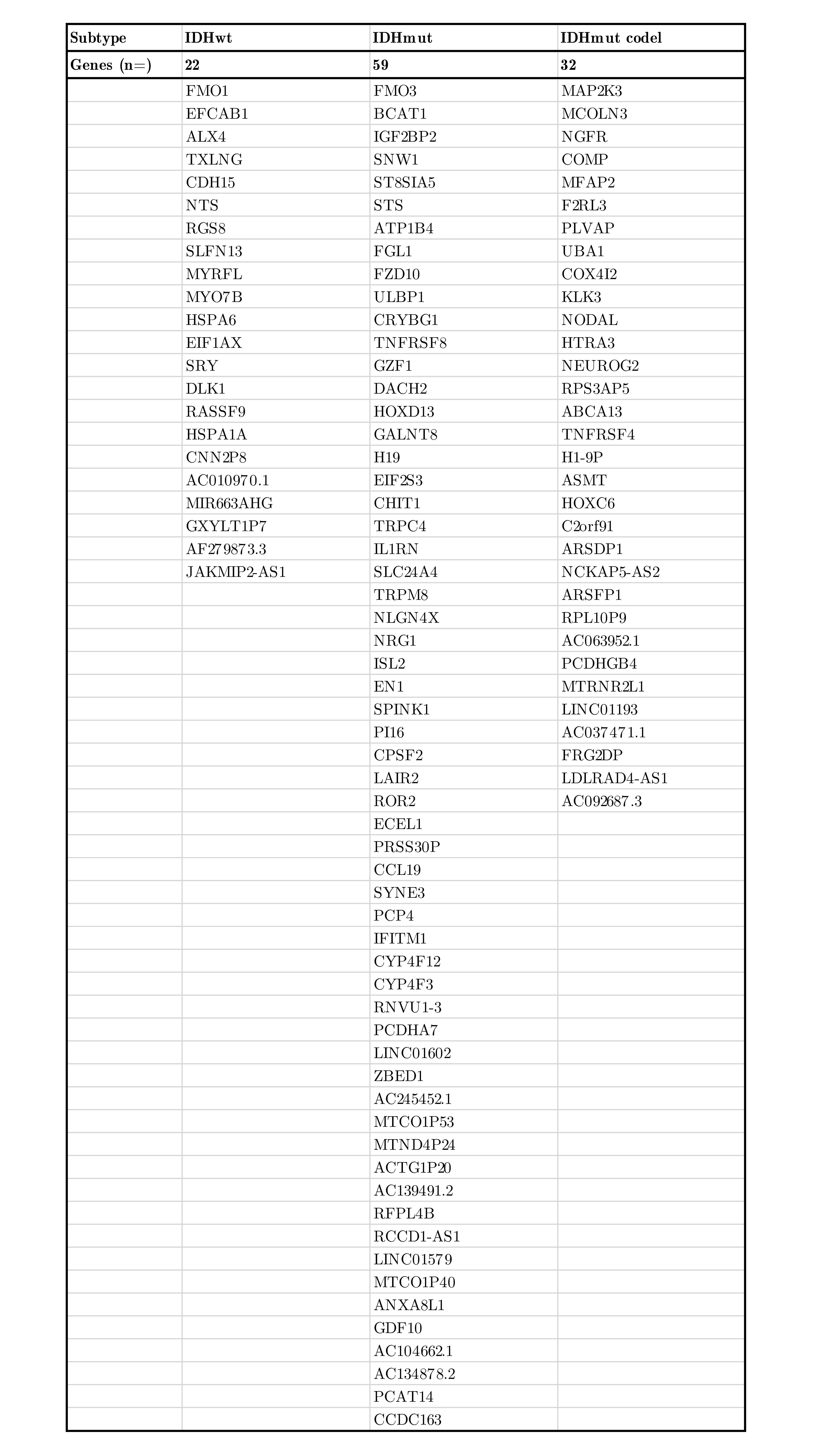

Supplement: Supplementary file 1 [file cancers-14-04114-s001.zip › Supplementary Table S5.png]
